# Effects of dietary L-Citrulline supplementation on growth performance, meat quality, and fecal microbial composition in finishing pigs

**DOI:** 10.3389/fmicb.2023.1209389

**Published:** 2023-08-03

**Authors:** Junhua Du, Mailin Gan, Zhongwei Xie, Chengpeng Zhou, Yunhong Jing, Menglin Li, Chengming Liu, Meng Wang, Haodong Dai, Zhiyang Huang, Lei Chen, Ye Zhao, Lili Niu, Yan Wang, Shunhua Zhang, Zongyi Guo, Linyuan Shen, Li Zhu

**Affiliations:** ^1^Key Laboratory of Livestock and Poultry Multi-omics, Ministry of Agriculture and Rural Affairs, College of Animal and Technology, Sichuan Agricultural University, Chengdu, China; ^2^Farm Animal Genetic Resource Exploration and Innovation Key Laboratory of Sichuan Province, Sichuan Agricultural University, Chengdu, China; ^3^Chongqing Academy of Animal Science, Chongqing, China

**Keywords:** L-Citrulline, growth performance, carcass characteristics, fecal microbes, finishing pigs

## Abstract

Gut microbiota play an important role in the gut ecology and development of pigs, which is always regulated by nutrients. This study investigated the effect of L-Citrulline on growth performance, carcass characteristics, and its potential regulatory mechanism. The results showed that 1% dietary L-Citrulline supplementation for 52 days significantly increased final weight, liveweight gain, carcass weight, and average backfat and markedly decreased drip loss (*p* < 0.05) of finishing pigs compared with the control group. Microbial analysis of fecal samples revealed a marked increase in α-diversity and significantly altered composition of gut microbiota in finishing pigs in response to L-Citrulline. In particular, these altered gut microbiota at the phylum and genus level may be mainly involved in the metabolic process of carbohydrate, energy, and amino acid, and exhibited a significant association with final weight, carcass weight, and backfat thickness. Taken together, our data revealed the potential role of L-Citrulline in the modulation of growth performance, carcass characteristics, and the meat quality of finishing pigs, which is most likely associated with gut microbiota.

## 1. Introduction

The intestinal microbiota is a complex and diverse ecosystem consisting of numerous microorganisms, which play a crucial role in supporting the nutritional, physiological, and immune functions of pigs (Brestoff and Artis, 2013; Fouhse et al., 2016). Pigs possess the capacity to harbor intricate microbial communities. The relationship between the host and symbiotic microbiota is characterized by interdependence and mutual influence. Moreover, the modulation of the microbiota can be employed to enhance the health and productivity of pigs (Yang et al., 2017). The composition of gut microbiota is typically influenced by various factors including host genotype, age, gender, environment, diet, and antibiotic usage. Rothschild et al. (2018) believed that the average heritability of gut microbiota is estimated to be merely 1.9%. Additionally, over 20% of the variation in microbial composition can be attributed to environmental factors such as diet, medications, and lifestyle choices. However, food is the most important factor among many factors affecting the composition of pig gut microbiota, which can explain 35% of the variation of pig gut microbiota composition (Wang et al., 2019). It was reported that dietary changes can cause changes in the cecal microbiota within 24 h (David et al., 2014). The beneficial impact of microbial community changes on the host primarily relies on the abundance and distinctiveness of the altered microorganisms, as well as the metabolites they produce. Currently, numerous dietary nutrients and functional additives, including probiotics, prebiotics, and enzymes, have been identified as effective regulators of intestinal microorganism composition in pigs, thereby promoting intestinal health.

Citrulline, also referred to as Carbamylornithine ornithine, exists in the L-type conformation, hence it is commonly known as L-Citrulline. Within the body, Citrulline (Cit) is primarily metabolized by arginine and nitric oxide (NO), leading to the production of NO and polyamines as its final products, respectively (Tsuboi et al., 2018). In numerous human studies, L-Citrulline has been utilized as a dietary supplement with the purpose of providing a substrate for the synthesis of arginine (ARG) or as a precursor for nitric oxide (NO) (Papadia et al., 2018). It was reported that L-Citrulline serves as an arginine precursor more productively than arginine itself (Schwedhelm et al., 2008). At present, although L-Citrulline has shown many beneficial effects, which can promote weight gain (Meesters et al., 2020), improve immunity (Cai et al., 2020), and decrease the proportion of senescent cells (Tsuboi et al., 2018), blood glucose and blood lipids (Danboyi et al., 2021), muscle wasting and augmenting exercise (Eshreif et al., 2020). However, the potential impact of dietary L-Citrulline supplementation on the growth and pork quality of finishing pigs is still unclear. Previously the studies suggested that the synthesis, breakdown, and metabolism of L-Citrulline primarily occur in the intestine, whereas, it remains unknown whether the mechanism of action of citrulline is associated with the host microbiota. In this study, we investigated the effects of dietary L-Citrulline supplementation on growth performance, carcass characteristics, and meat quality as well as gut microbiota, and identified its correction between phenotypes and gut microbiota in finishing pigs. The results provide novel insights into the biological potential of L-Citrulline in improving pigs with significant nutritional values.

## 2. Materials and methods

### 2.1. Animals and experimental design

A total of 12 large white pigs with 72.25 ± 4.30 kg provided by a pig breeding company in Sichuan Province, China, were randomly allocated to two different diet groups (*n* = 6 per group) including (1) a basal diet or (2) a basal diet and 1% L-Citrulline (10 g L-Citrulline per 1 kg liquid feed), respectively. We used a commercial feed from Cargill Feed (Chongqing) Co., Ltd. as basal feed for all finishing pigs. The composition and nutrient levels of the basal diet are given in Supplementary Table S1. All pigs were given free access to feed and water, and housed under similar environmental conditions with ambient temperatures around 25–35°C. Using the pig individual weighing device D600 (Jiangsu Kono Animal Husbandry Equipment Technology Co., Ltd.), the initial and final body weights of finishing pigs were measured individually before and after feeding experiments. L-Citrulline with a minimum purity of 98% was purchased from Shanghai Yuanye Biological Co., Ltd. All experimental procedures were approved by the Animal Ethical and Welfare Committee of Sichuan Agricultural University, Chengdu, China (approval number DKY-B20131403).

### 2.2. Animal analysis and sample collection

We collected fresh feces from finishing pigs, and immediately froze and stored them at −80°C for DNA extraction, at day 52 post-treatment. Subsequently, carcass characteristics were further evaluated. Briefly, all pigs were moved to a commercial slaughter room and electrically stunned, and then slaughtered according to standard commercial procedures. The live body weight and hot carcass weight were immediately recorded for the dressing percentage calculation. Meanwhile, carcass length also was recorded. Backfat thickness (mm) was calculated by averaging the scores of three regions at the first rib, last rib, and last lumbar vertebrae of the right carcass sides. The eye muscle area was measured at the last rib using vernier calipers. The pH_45 min_, meat color, drip loss, purge loss, and cook loss measurements were conducted using the longissimus dorsi (LD) muscle obtained from the left side of each carcass.

### 2.3. Meat quality measurement

The pH and meat color parameters (L* lightness, a* redness, and b* yellowness) were assessed 45 min post-slaughter using a pH meter (pH-STAR, MATTHAUS, Germany) and a portable chromameter (CR-400, KONICA MINOLTA, Japan) respectively. Three measurements were taken at different areas of each chop, and the average value was calculated. Drip loss was assessed by weighing approximately 25 g of muscle after a 45-min postmortem period (W1). The muscle was then placed in a storage bag attached to a fishhook and kept at 4°C for 24 h. Afterward, the muscle was removed from the fishhook, carefully dried, and reweighed (W2). The drip loss value was calculated using the following formula: drip loss (%) = (W1 − W2)/W1 * 100. To determine the cooking loss, approximately 100 g of muscle was weighed (W3) and cooked in a steamer for 30 min. Following the cooking process, the muscle sample was promptly removed from the steamer, allowed to cool for 20 min at room temperature, and reweighed (W4). The cooking loss was calculated using the formula: cooking loss (%) = (W3 − W4)/W3 * 100. Purge loss was measured and calculated according to the method described by Setyabrata and Kim (2019).

### 2.4. 16S rRNA gene sequencing

The CTAB/SDS method was used to extract the total genome DNA in samples (Sambrook et al., 1989). DNA concentration and purity were monitored on 1% agarose gel. According to the concentration, DNA was diluted to 1 ng/μL with sterile water. Then, the diluted genomic DNA was used as a template for PCR amplification. The hypervariable V3-V4 region of the 16S rRNA gene was amplified using a specific primer pair: 341F (CCTAYGGGRBGCASCAG) and 806R (GGACTACNNGGGTATCTAAT). PCR amplification was performed using Phusion^®^ High-Fidelity PCR Master Mix (New England Biolabs, Ipswich, MA, United States) following the manufacturer’s instructions. Finally, the PCR products were detected by 2% agarose gel electrophoresis.

### 2.5. Bioinformatics analysis of sequencing data

According to the barcode sequence and PCR amplification primer sequence, the sample data were separated from the offline data. After the barcode and primer sequences were truncated, the reads of the samples were spliced using FLASH (V1.2.11)^1^ (Magoc and Salzberg, 2011) software to obtain Raw Tags. Subsequently, fastp software was used to perform quality control on the obtained Raw Tags to obtain high-quality Clean Tags. Finally, Vsearch software is used to compare Clean Tags with the database to detect chimeras and remove them, so as to obtain the final valid data, Effective Tags (Haas et al., 2011).

For the Effective Tags obtained above, the DADA2 module in QIIME2 software is used for noise reduction, and the sequences with an abundance of less than 5 are filtered out to obtain the final ASVs (Amplicon Sequence Variants) and the characteristic table.

QIIME2 software was used to calculate the observed_ASVs and chao1 indexes to analyze the diversity of fecal microbial composition and draw a rarefaction curve. Principal component analysis was used to analyze the β diversity between different groups (packages “vegan”) to compare the differences in fecal microbial composition under different treatments. In order to obtain the different species between different groups, LEfSe software was used to analyze the significant difference in species between different groups under different treatments, and LDA linear discriminant (LDA Score) was used to quantify the effect of different species on the difference between groups. Furthermore, ASVs functions were annotated against the Greengenes database using PICRUST (Haas et al., 2011). Finally, Pearson correlation analysis was used to determine the correlations of the top 40 phyla and genera with significant differences in relative abundance from final weight, carcass weight, and average backfat.

### 2.6. Statistical analyses

Growth performance, carcass characteristics, and relative abundance of fecal microbes of finishing pigs were analyzed by SPSS 22.0 software. Differences between mean values were evaluated by independent sample *T*-test. Significance was set at a *p-*value of 0.05.

## 3. Results

### 3.1. Effect of dietary L-Citrulline on growth performance and carcass characteristics

To investigate the effect of L-Citrulline on the growth performance and carcass weight of finishing pigs, large white pigs without difference in both initial weight and age were supplemented with 1% L-Cit for 52 days. As shown in Table 1, we detected a marked increase in the final weight of pigs fed with L-Citrulline (L-Cit) compared with the control group (NC), which may be due to significantly increased average daily gain (ADG) upon supplementation with L-Citrulline. In addition, supplementation with L-Citrulline resulted in remarkably increased carcass weight by 7.9% without affecting carcass length in finishing pigs. No difference was determined in eye muscle area between the two groups, whereas pigs fed with L-Citrulline exhibited significantly higher backfat thickness when compared to the basal diet-fed pigs.

**Table 1:** Effect of dietary L-Citrulline supplementation on growth performance and carcass characteristics of finishing pigs.

Item	Group		*p*-value
	NC (*n* = 6)	L-Cit (*n* = 6)	
Initial weight, kg	71.75 ± 4.51	72.75 ± 4.44	0.71
Final weight, kg	112.33 ± 12.96	126.83 ± 4.02	<0.05
ADG, kg/day	0.78 ± 0.18	1.04 ± 0.13	<0.05
Initial age, day	151.17 ± 7.52	155.50 ± 3.29	0.23
Carcass weight, kg	85.08 ± 8.85	96.42 ± 2.35	<0.05
Dressing percentage, %	75.85 ± 0.02	76.04 ± 0.01	0.85
Carcass length, cm	102.58 ± 3.14	105.08 ± 2.54	0.16
Average backfat, cm	1.52 ± 0.25	1.82 ± 0.22	<0.05
Eye muscle area, cm^2^	47.20 ± 8.18	46.46 ± 9.55	0.89

NC, control group; L-Cit, L-Citrulline group; ADG, average daily gain.

### 3.2. Effect of dietary L-Citrulline on meat quality of finishing pigs

Next, we accessed the effect of dietary L-Citrulline on the meat quality of finishing pigs. As shown in Table 2, drip loss significantly decreased from 4.33 to 2.32% after dietary L-citrulline supplementation. However, no significant differences were observed in pH_45 min_, meat color, purge loss, and cook loss between the two groups.

**Table 2:** Effect of dietary L-Citrulline supplementation on meat quality of finishing pigs.

Item	Group		*P*-value
	NC (*n* = 6)	L-Cit (*n* = 6)	
pH_45 min_	6.27 ± 0.32	6.03 ± 0.10	0.11
L*_45 min_	44.74 ± 1.27	44.08 ± 2.02	0.52
a*_45 min_	3.96 ± 1.07	5.11 ± 1.19	0.11
b*_45 min_	2.95 ± 0.74	3.31 ± 0.70	0.41
Purge loss, %	33.40 ± 0.09	31.42 ± 0.04	0.63
Drip loss, %	4.33 ± 0.01	2.32 ± 0	<0.05
Cook loss, %	33.76 ± 0.02	34.82 ± 0.01	0.27

### 3.3. Altered fecal microbes in finishing pigs

**Figure 1:**
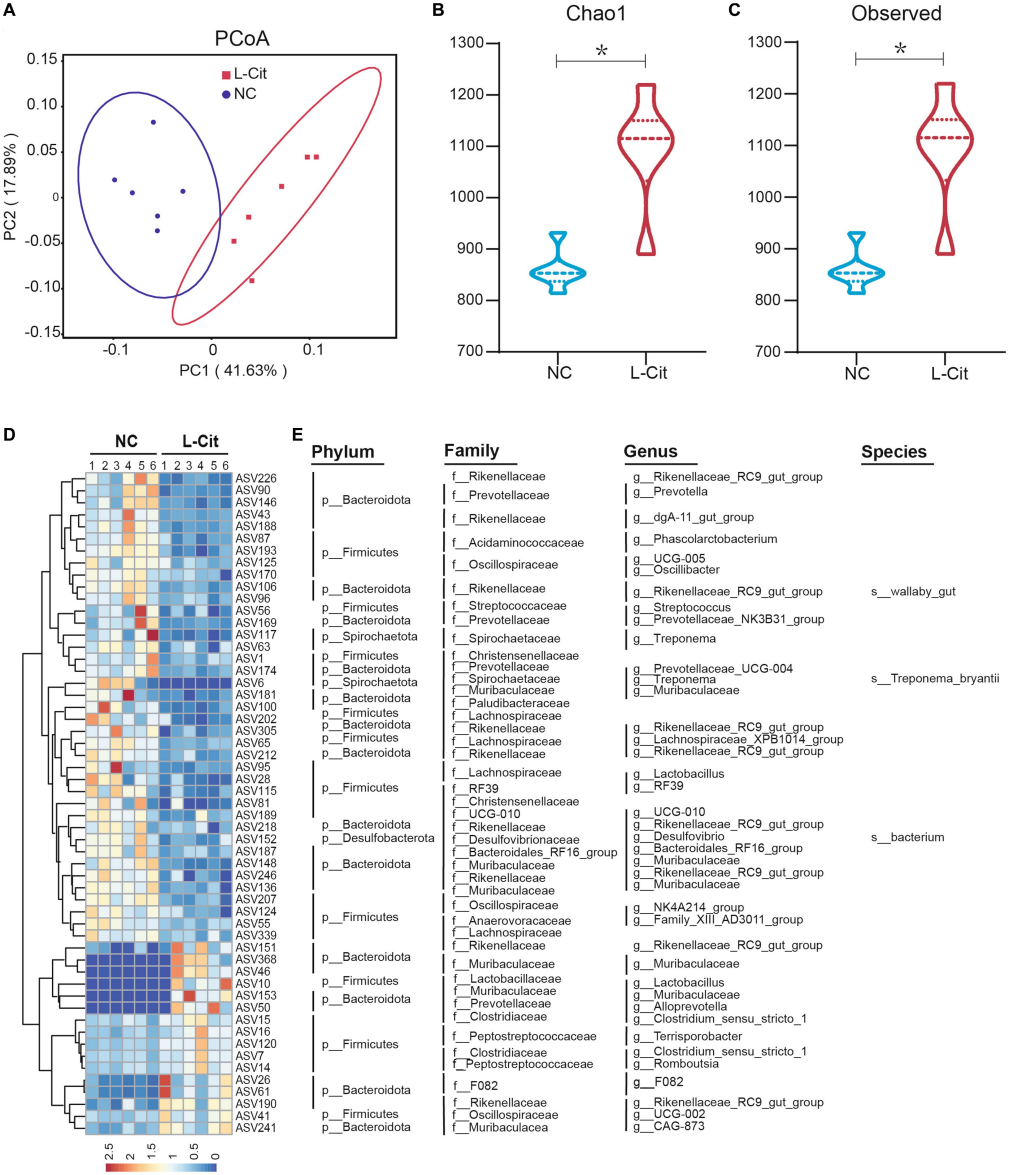
Number of amplicon sequence variants (ASVs) and the richness and diversity of the fecal microbiota in finishing pigs. **(A)** Principal coordinate analysis. **(B)** Chao1 index. **(C)** Observed index. **(D)** Heatmap showing the relative abundance of bacterial ASVs in the top 55. **(E)** Represented bacterial taxa information (phylum, family, genus, and species) of 55 ASVs from **(D)** are shown. *p < 0.05.

Emerging evidence reveals a potential role of gut microbiota in nutrients-mediated alterations of growth performance and carcass characteristics, and L-Citrulline may contribute to improving gut health. To characterize the regulatory mechanism by which L-Citrulline affected growth performance and carcass characteristics of finishing pigs, we analyzed gut microbiota by performing 16S rRNA gene sequencing on fecal samples collected from L-Cit and NC groups, respectively. After quality control, 4,066 valid ASVs were generated for each sample to perform for subsequent analysis, which was clustered at 100% similarity. As shown in Supplementary Figure S1, analysis of the dilution curve suggested that sequencing depth covered rare new phylotypes and most of the diversity. Weighted UniFrac-based principal coordinates analysis (PCoA) revealed a distinct clustering of microbiota composition in each treatment group (Figure 1A). To gain more insight into alterations of gut microbiota in finishing pigs upon L-Cit, α-diversity analysis was further performed. As shown in Figures 1B,C, the L-Cit group exhibited a higher diversity of microbiota as evidenced by increased indexes of Chao1 and Observed when compared with the control group, as well as determined by Ace index analysis (Supplementary Figure S2).

To better understand the effect of L-Citrulline on gut microbiota in finishing pigs, we next identified the specific bacterial phylotypes responding to L-Citrulline after deleting data with extremely low abundance among fecal samples. L-Citrulline dramatically altered 199 amplicon sequence variants (ASVs), among which 124 decreased and 75 increased in finishing pigs after L-Citrulline supplementation (Supplementary Table S2). Here, we mainly focused on the top 55 altered ASVs abundance of gut microbiota in response to L-Citrulline, among which 39 decreased and 16 increased in finishing pigs after L-Citrulline supplementation (Figure 1D). Notably, most of these ASVs were mainly classified in the Rikenellaceae and Lachnospiraceae families (Figure 1E), which are two important families involved in glucose metabolism, lipid metabolism, and skeletal muscle (Wu et al., 2021; Du et al., 2022). Taken together, these results suggested that L-Citrulline may significantly alter the gut microbiota of finishing pigs.

### 3.4. Alterations of fecal microbes at the phylum and genus level

**Figure 2:**
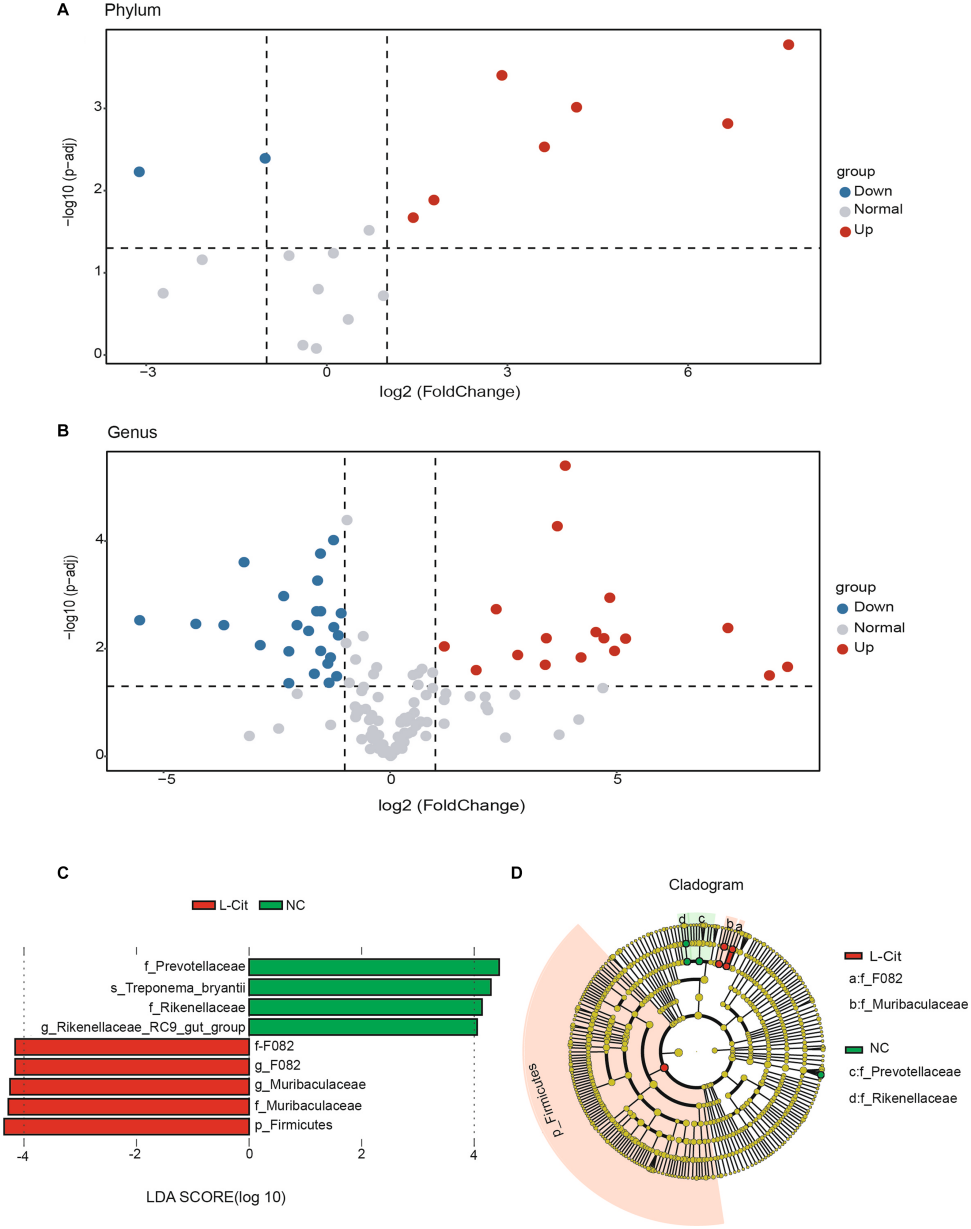
Screening of fecal microbial biomarkers in the groups. **(A,B)** Differential volcano map [x-axis coordinate, log2 fold change (FC); y-axis coordinate, adjusted *p-*value]. Each point in the graph represents a phyla or genera and the two lines parallel to the y axis represent FC = 1 and FC = −1. The dotted line parallel to the x axis represents 2log10 (0.05), and the points above, left and right the dotted line represents phyla or genera with significance at P, 0.05. **(C)** Histogram of the results of LEfSe among the L-Cit group and the control group and their respective effect sizes. **(D)** Cladogram showing taxonomic representation of differences among the L-Cit group and the control group.

We then analyzed the fecal microbiota composition at both phylum and genus levels of finishing pigs under different diet conditions. The volcano plots analysis showed that 7 phyla significantly increased and 2 significantly decreased in finishing pigs upon supplementation with L-Citrulline (Figure 2A). Similarly, L-Cit pigs exhibited 17 significantly increased genera and 23 significantly decreased genus (Figure 2B). Based on the results of the volcano map, we employed the LEfSe algorithm analysis (LDA log score threshold ≥4) to identify high-dimensional biomarkers among two diet groups. A total of 9 potential biomarkers were observed in the two groups, in which f_F082, g_F082, g_Muribaculaceae, f_Muribaculaceae, and p_Firmicutes showed in L-Cit group, while f_Prevotellaceae, s_Treponema_bryantii, f_Rikenellaceae, and g_Rikenellaceae_RC9_gut_group found in NC group (Figure 2C). Of note, at the phylum level, Firmicutes is the most differentially abundant taxon in the L-Cit group (Figure 2D). Therefore, these key phylotypes may contribute to the differences in microbiota composition in the groups.

### 3.5. Analysis of top 10 abundant phylum and genus

**Figure 3:**
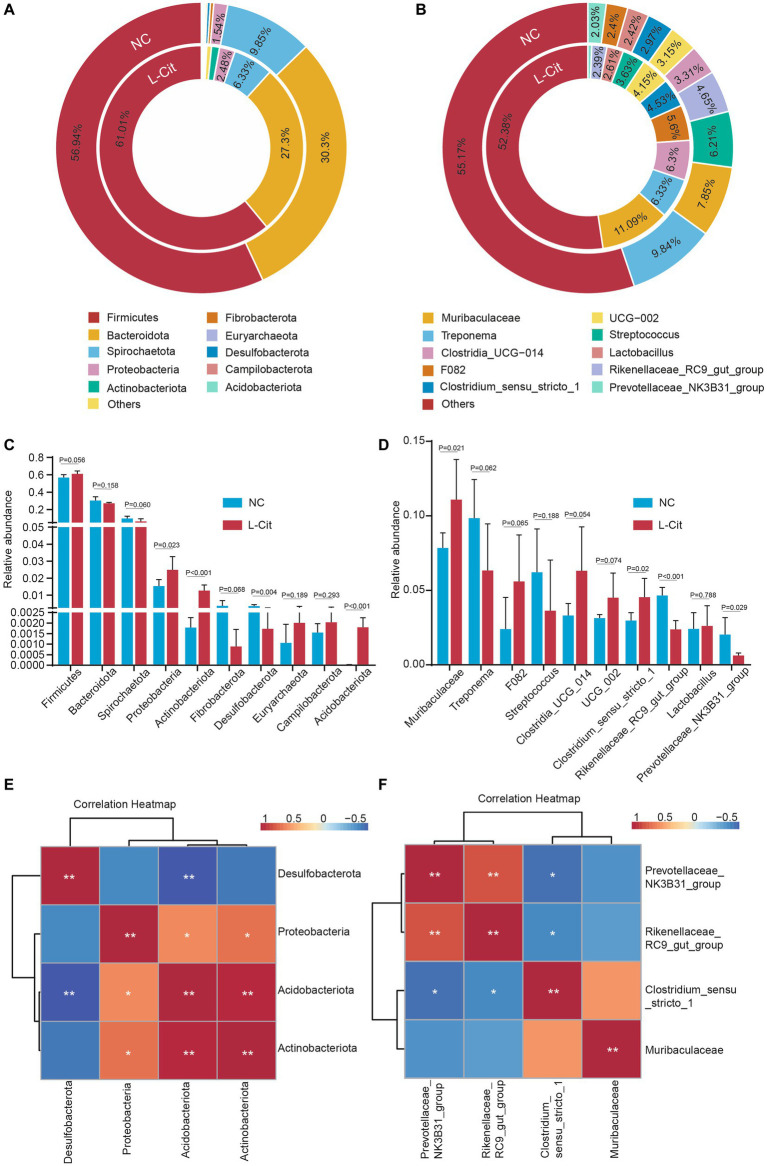
Composition of and differences in fecal microbiota at the phylum level and genus level. **(A)** Microbial composition of the feces at the phylum level. **(B)** Microbial composition of the feces at the genus level. The inner circle is the distribution of the fecal microbiota in the L-Cit group, and the outer circle is the distribution of the fecal microbiota in the control group. **(C)** Relative abundance of differential phyla compared between groups. **(D)** Abundance of differential genera compared between groups. **(E)** Spearman correlation analysis of four significant phyla. **(F)** Spearman correlation analysis of four significant genera. *p < 0.05, **p < 0.01.

Given the importance of relatively abundant gut microbiota in the regulation of gut ecology and host health, we further focused on the top 10 abundant phylum and genus. At the phylum level, Firmicutes and Bacteroidetes were the most abundant phylum in the two groups, accounting for about 85–90% (Figure 3A), which is consistent with previous findings of gut microbiota in pigs and humans. Relative to significantly altered Actinobacteriota, Desulfobacterota, Proteobacteria, and Acidobacteriota are among the top 10 microbial abundances of the gut microbiotas at the phylum level (Figure 3C). However, the three most abundant phyla of bacteria including Firmicutes, Bacteroidota, and Spirochaetota did not differ in pigs fed with L-Citrulline from the control pigs (Figure 3C). Given previous studies showing an obvious association between animal metabolism and the ratio of Firmicutes: Bacteroidetes (F:B), we also examined the F:B ratio between the two groups. As shown in Supplementary Figure S3, the correlation analysis showed that F:B ratio had no correlation with growth performance and carcass characteristics such as final weight, carcass weight, and average backfat, although the F:B ratio slightly increased from 1.92 in NC group to 2.24 in L-Cit group.

At the top three microbial abundances of the gut microbiota at genus level, Muribaculaceae and Treponema were the two most abundant genera between the two diet groups, while Streptococcus and Clostridia_UCG-014 were especially shown in the NC group and L-Cit group, respectively (Figure 3B). To reveal the difference at genus levels in finishing pigs upon L-Citrulline, we analyzed the top microbial abundances of the gut microbiota. As shown in Figure 3D, supplementation with L-Citrulline significantly increased the relative abundance of Muribaculaceae and Clostridium_sensu_stricto_1, but remarkably reduced the relative abundance of Rikenellaceae_RC9_gut_group and Prevotellaceae_NK3B31_group in finishing pigs. In addition, to better understand gut microbiota alterations responding to L-Citrulline, spearman correlation analysis was performed to determine the correlations of significant phyla and genera. Based on abundance at the phylum level, Actinobacteriota was positively associated with Acidobacteriota and Proteobacteriota, and Acidobacteriota, respectively, showed a significantly negative or positive correction with Desulfobacterota, or Proteobacteriota (Figure 3E). At the genus level, there was a significantly negative association between Prevotellaceae_NK3B31_group, Rikenellaceae_RC9_gut_group, and Clostridium_sensu_stricto_1 (Figure 3F). Increased Prevotellaceae_NK3B31_group may contribute to Rikenellaceae_RC9_gut_group but not Clostridium_sensu_stricto_1 (Figure 3F).

### 3.6. Altered gut microbiota function in finishing pigs upon L-Citrulline

**Figure 4:**
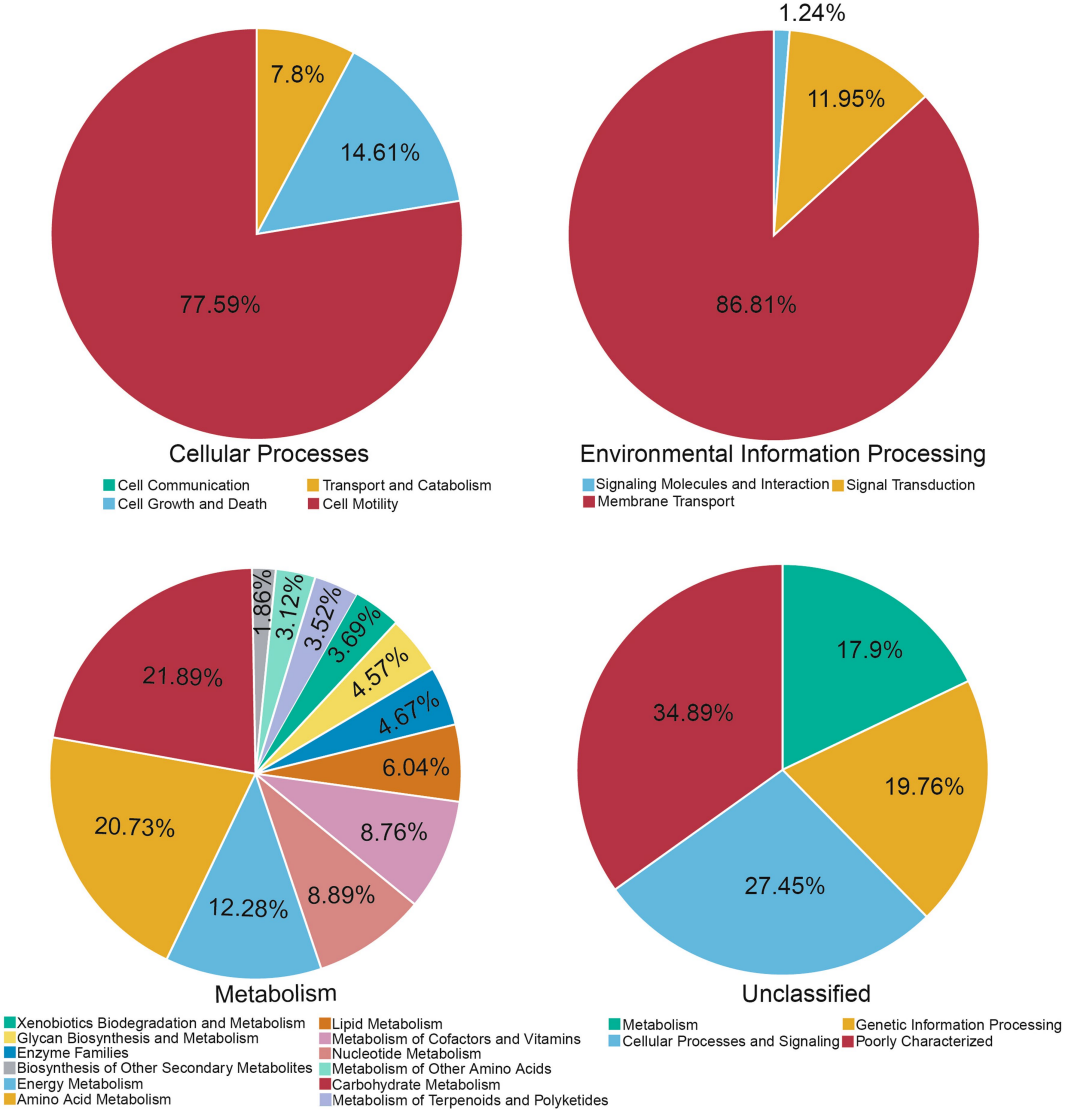
Proportion of the number of non-redundant genes assigned to KEGG pathways.

To gain more insight into the effect of L-Citrulline on gut microbiota, we investigated the potential function of gut microbiota responding to L-Citrulline in finishing pigs by blasting Unigenes to the KEGG databases. Firstly, we analyzed the number of non-redundant genes assigned to KEGG pathways (Supplementary Figure S4). Because the number of non-redundant genes assigned to human diseases and organismal systems was relatively less compared with other pathways, we mainly focused on other KEGG pathways. As shown in Figure 4, most genes were assigned to cell motility, membrane transport, carbohydrate metabolism, energy metabolism, carbohydrate metabolism, and amino acid metabolism and were poorly characterized.

**Figure 5:**
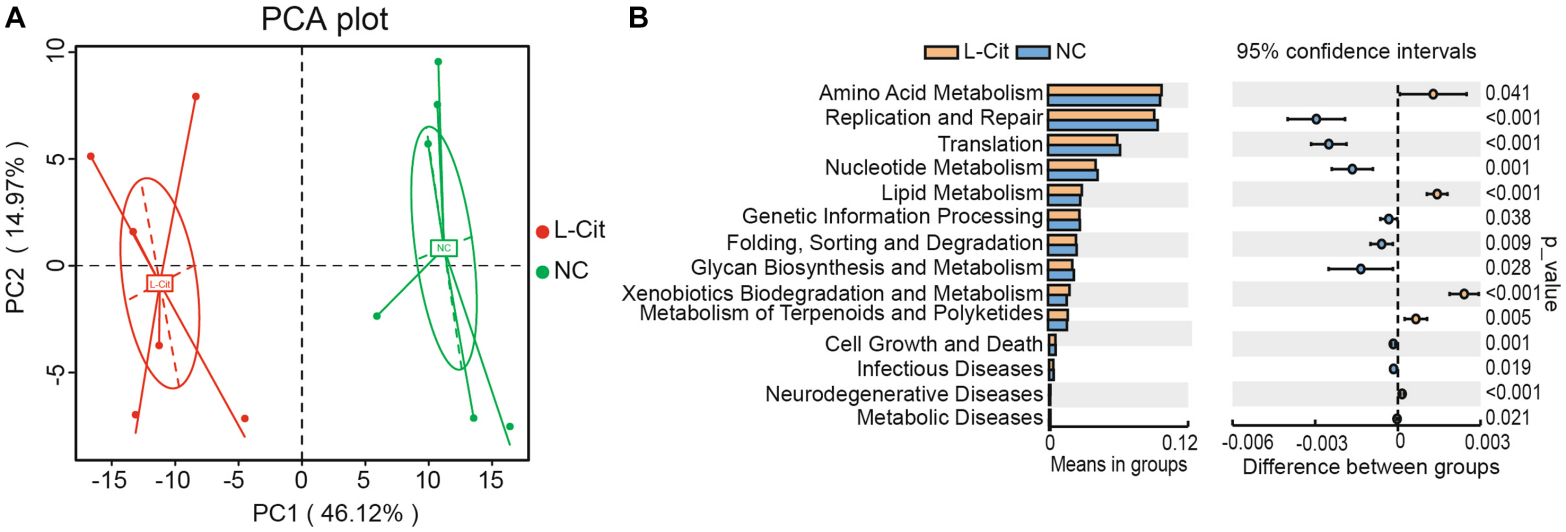
Prediction of metabolic pathways regulated by fecal microbes. **(A)** Principal component analysis. **(B)** *T*-test analysis.

Subsequently, predictive function richness was used to generate a principal component analysis (PCA) plot. Samples in the L-Cit group and control group were clustered separately (Figure 5A). Then, a T-test analysis was carried out on the abundance of annotated KEGG levels (level 2) of fecal microbiota composition of finishing pigs in the groups. In total, 5 metabolic pathways increased significantly in the L-Cit group compared with the control group, including amino metabolism, lipid metabolism, xenobiotics biodegradation, and metabolism, metabolism of terpenoids and polyketides, and neurodegenerative disease, and 9 metabolic pathways decreased significantly in the L-Cit group compared with the control group, including those of nucleotide metabolism, glycan biodegradation and metabolism and metabolic disease (Figure 5B).

### 3.7. Correlation analysis between fecal microbiota and final weight, carcass weight, average backfat, and drip loss

**Figure 6:**
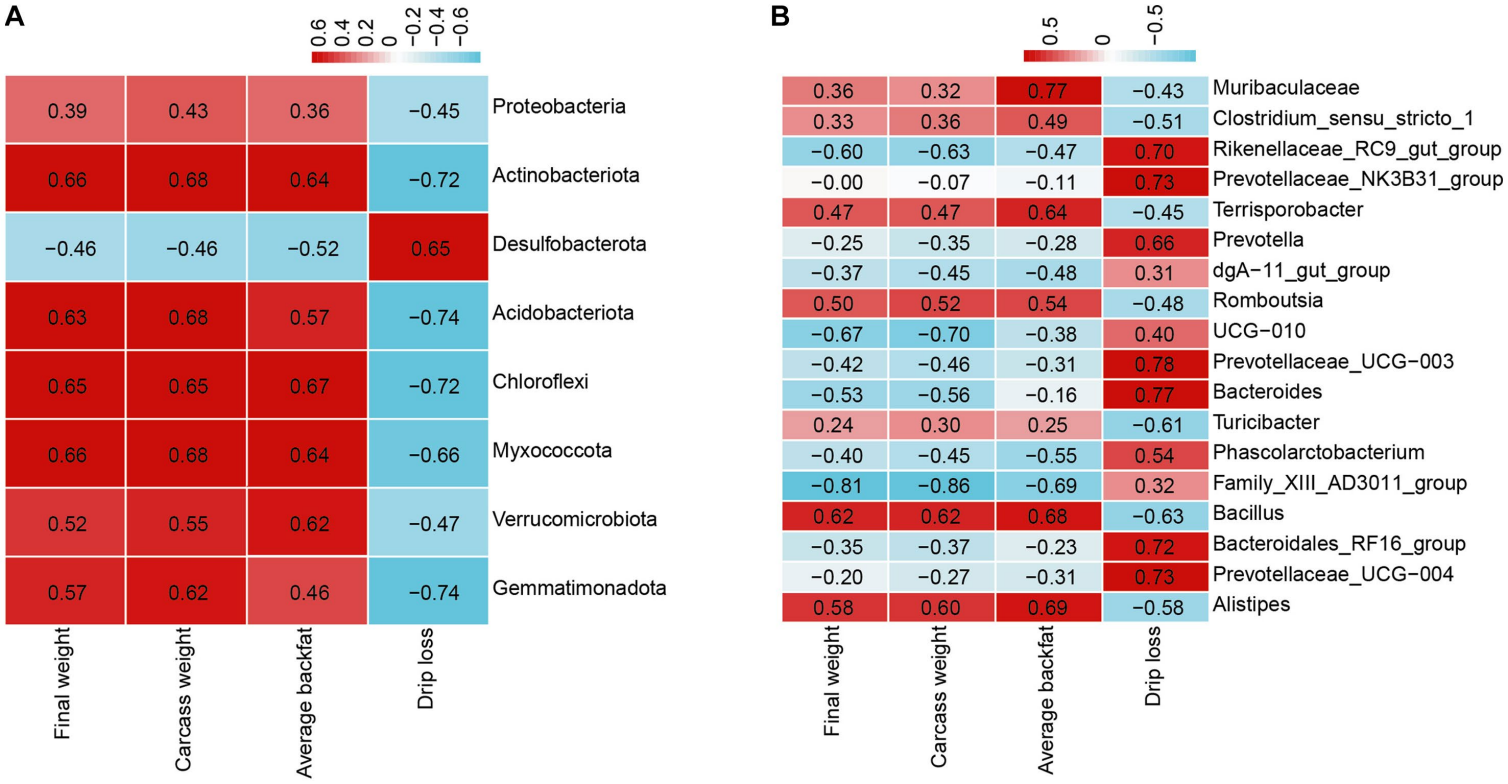
Pearson correlation analysis between fecal microbiota and final weight, carcass weight, average backfat, and drip loss. **(A)** Correlation analysis between the significant relative abundance of the top 40 phyla and final weight, carcass weight, average backfat, and drip loss. **(B)** Correlation analysis between the significant relative abundance of the top 40 genera and final weight, carcass weight, average backfat, and drip loss.

To gain more insight into the effect of L-Citrulline on finishing pigs, we examined the correlations of the 40 most significantly abundant phyla and genera in fecal microbiota with growth performance, carcass traits, and meat quality. The Pearson correlation analysis indicated the significant correlations between fecal microbe and final weight, carcass weight, average backfat, and drip loss. As shown in Figure 6A, average backfat, carcass weight, and as well as final weight were positively associated with the most significant phyla, including Actinobacteriota, Acidobacteriota, Chloroflexi, Myxococcota, Verrucomicrobiota, and Gemmatimonadota, and were negatively correlated with Desulfobacterota. The result of drip loss was just the opposite. At the genus levels, final weight, carcass weight, and average backfat were positively correlated with Alistipes, Bacillus, and Romboutsia, while were negatively correlated with Family_XIII_AD3011_group (Figure 6B). Moreover, average backfat was also positively correlated with Terrisporobacter and Muribaculaceae (Figure 6B). Drip loss were significantly positively correlated with Rikenellaceae_RC9_gut_group, Prevotellaceae_NK3B31_group, Prevotella, Prevotellaceae_UCG-003, Bacteroides, Bacteroidales_RF16_group, and Prevotellaceae_UCG-004, while were significantly negative connected with Turicibacter and Bacillus (Figure 6B). These results indicated the interaction of microbes with final weight, carcass weight, average backfat, and drip loss.

## 4. Discussion

L-Citrulline serves as a highly effective precursor for the synthesis of arginine, an essential amino acid crucial for normal growth and development in poultry. Both under normal conditions and during heat stress, arginine plays a pivotal role in promoting poultry’s body weight, carcass weight, and muscle development (Kalvandi et al., 2022). Furthermore, the supplementation of L-Citrulline has been found to have protective effects on the growth performance of broilers under heat stress conditions. It aids in reducing protein catabolism, thereby preserving muscle mass and promoting better growth outcomes (Uyanga et al., 2022). In this study, dietary L-Citrulline supplementation significantly increased the final weight and carcass weight of finishing pigs. The final weight, total weight gain, and carcass weight of finishing pigs in the L-Cit group were 7.90, 16.50, and 7.91% higher than those in the control group, respectively. The results indicated that dietary L-Citrulline supplementation promoted weight gain and profitability in finishing pigs. However, the precise mechanism by which L-Citrulline regulates weight gain in this context remains uncertain. It has been suggested that this effect may be attributed to the conversion of L-Citrulline into L-arginine, which plays a role in intestinal protection (Kobata et al., 2007). It is worth noting that the gastrointestinal tract’s health significantly impacts nutrient digestion, absorption, and subsequently, animal growth and development. Additionally, nitric oxide is known to play a crucial role in maintaining nutrient metabolism, particularly in sugar, fat, protein, and amino acid metabolism (Jobgen et al., 2006). Hence, it is plausible that citrulline may enhance nitric oxide metabolism and nutrient transport within the body, thereby promoting weight gain in finishing pigs. Further research is needed to fully elucidate the mechanisms involved in the observed effects.

It is well known that pH, meat color, and water holding capacity are key indicators that reflect the taste and visual appeal of meat, which are also crucial factors influencing meat quality and consumer preferences (Popp et al., 2015; Ramanathan et al., 2020). In this study, although there was no statistically significant difference in the pH value between the two dietary groups, all observed values fell within the normal range outlined by the current national objective standard for pork eating quality (NY/T2793-2015), suggesting that a short-term inclusion of citrulline in the diet may have no significant effect on the pH value of finishing pigs. Notably, the L-Cit group exhibited a significant reduction in meat drip loss compared to the control group, implying that the addition of citrulline in the diet may help mitigate water loss, and enhance meat quality.

The gut microbiota serves as a vital link between diet and host health, playing a crucial role in shaping the structure of the microbial community within the gut. The composition of the gut microbiota is significantly influenced by dietary factors. Through its intricate interactions with the host, the gut microbiota exerts a profound influence on various aspects of host physiology, including the maintenance of homeostasis, organ development, metabolic processes, and immune response (Fouhse et al., 2016; Shang et al., 2022). According to the PCoA result, the two groups were clearly separated, suggesting significant influences in bacterial composition among the L-Cit group. It was reported that microbial diversity serves as a reliable indicator of gut health, with higher α diversity indicating a more intricate and resilient composition of the intestinal microbiota (Sommer et al., 2017). Increased microbial diversity signifies enhanced resistance to external disruptions, greater adaptability, and improved capacity for self-restoration, all of which are beneficial to the host. Consequently, a diverse gut microbial community is considered advantageous for maintaining optimal gut function and overall well-being (Sommer et al., 2017; Lang et al., 2018). The α diversity measurements in finishing pigs receiving dietary L-Citrulline supplementation suggested that the presence of a greater variety of species within their gut microbiota could potentially enhance their resilience to environmental factors. This can be attributed to the compensatory effect exhibited by functionally related microorganisms within a well-balanced ecosystem, where the absence of certain species can be compensated by the functions performed by other microbial species.

The phyla Firmicutes and Bacteroidetes, which are commonly found in the gut microbiome of mammals, including pigs, have been strongly associated with energy metabolism and feed efficiency (Bergamaschi et al., 2020). The phylum Bacteroidetes has been found to exhibit higher abundance in pigs characterized by high feed efficiency or lean body composition, in contrast to pigs with lower feed efficiency or obesity (Jami et al., 2014). In this study, no significant difference in the abundance of these 2 taxa was observed between L-Cit and NC groups, Firmicutes and Bacteroidetes predominated in the fecal microbiome of all samples, and the relative abundance of Bacteroidetes in the fecal microbiome of the control group was slightly higher than in the L-Cit group. At the phylum level， Firmicutes was both core and differential bacteria of the L-Cit group and therefore may have important roles. The Prevotellaceae NK3B31 group, a subgroup within the genus Prevotella, has been identified to possess growth-promoting properties in pigs (Yang et al., 2017). However, our study indicated that the relative abundance of the control group was more compared with the L-Cit group. Muribaculaceae has been established to possess the ability to inhibit the development of inflammatory and metabolic diseases (Tavella et al., 2021). At the genus level, the abundance of Muribaculaceae in the L-Cit group was significantly increased compared with the control group, which indicated that Long-term consumption of L-Citrulline may contribute to the shape of disease-resistant characterization of the gut bacteria in finishing pigs. Furthermore, the Rikenellaceae_RC9_gut_group is known to possess the capability to degrade complex carbohydrates such as indigestible oligosaccharides, cellulose, hemicellulose, and resistant starch. As a result of this carbohydrate metabolism, they produce beneficial short-chain fatty acids such as acetate, butyrate, and lactic acid. It can be inferred that the presence of Rikenellaceae_RC9_gut_group in the gut microbiota of finishing pigs fed with L-Citrulline may enhance the digestion and absorption of nutrients in the host. By efficiently breaking down complex carbohydrates and producing beneficial metabolites, these bacteria can contribute to improving the host’s nutrient utilization and overall metabolic efficiency.

Finally, microbial functional analysis (PICRUSt) showed that a higher abundance of predicted functions related to amino acid metabolism, lipid metabolism, xenobiotics biodegradation, and metabolism and metabolism of terpenoids and polyketides was found in the L-Cit group, which may indicate that finishing pigs of dietary L-Citrulline supplementation gut contains more bacteria with greater metabolic capacity, and these bacteria may produce more energy for the hosts. It is notable that the L-Cit group only enriched in neurodegenerative disease, while the control group enriched in infectious diseases and metabolic diseases, which further indicated finishing pigs of dietary L-Citrulline supplementation may contribute to the shape of disease-resistant characterization of the gut bacteria in finishing pigs. The Pearson correlation analysis in our study showed that Rikenellaceae_RC9_gut_group was negatively correlated with carcass weight and final weight, suggesting that the low carcass weight and final weight in the control group may be attributed to the increased abundance of Rikenellaceae_RC9_gut_group.

## 5. Conclusion

In conclusion, dietary L-Citrulline supplementation had a significant effect on growth performance, and drip loss, as well as modulating the fecal microbiota of finishing pigs. Therefore, these results indicated that L-Citrulline could be one potential amino acid ingredient for finishing pigs, and whether the recommended addition proportion in the finishing is 1% remains to be confirmed by further research.

## Data availability statement

The data presented in the study are deposited in the NCBI repository, accession number CRA011705.

## Ethics statement

The animal study was reviewed and approved by the Ethics Committee of Sichuan Agricultural University.

## Author contributions

JD and MG: conceptualization and writing—original draft preparation. JD, LN, and ZH: methodology. ZX, CL, and HD: software. CZ, MW, and ML: validation. JD: formal analysis. YW: investigation. LC and SZ: resources. JD and ZX: data curation. LZ and LS: writing—review, editing, and funding acquisition. YZ and YJ: visualization. ZG, LZ, and LS: supervision. LZ: project administration. All authors contributed to the article and approved the submitted version.

## Funding

This work was supported by National Key Research and Development Program of China (2021YFD1200801); Sichuan Science and Technology Program (2021ZDZX0008, 2021YFYZ0030, scsztd-2023-08-09); The Earmarked Fund for CARS (CARS-pig-35); National Center of Technology Innovation for Pigs.

## Conflict of interest

The authors declare that the research was conducted in the absence of any commercial or financial relationships that could be construed as a potential conflict of interest.

## Publisher’s note

All claims expressed in this article are solely those of the authors and do not necessarily represent those of their affiliated organizations, or those of the publisher, the editors and the reviewers. Any product that may be evaluated in this article, or claim that may be made by its manufacturer, is not guaranteed or endorsed by the publisher.

## Footnotes

^1^ http://ccb.jhu.edu/software/FLASH/
