# Supplementary material for: Effects of dietary L-Citrulline supplementation on growth performance, meat quality, and fecal microbial composition in finishing pigs
Source: Front Microbiol. 2023 Aug 3;14:1209389. doi: 10.3389/fmicb.2023.1209389 (PMC10442155; doi:10.3389/fmicb.2023.1209389)
Supplement: Supplementary file 1 [file Supplementary_Figure.docx]

Effects of Dietary L-Citrulline Supplementation on Growth Performance and Fecal Microbial Composition in Finishing Pigs

**Junhua Du^1,2,†^, Mailin Gan^1,2,†^, Zhongwei Xie^1,2^, Chengpeng Zhou^1,2^, Yunhong Jing^1,2^, Menglin Li^1,2^, Chengming Liu^1,2^, Meng Wang^1,2^, Haodong Dai^1,2^, Zhiyang Huang^1,2^, Lei Chen^1,2^, Ye Zhao^1,2^, Lili Niu^1,2^, Yan Wang^1,2^, Shunhua Zhang^1,2^, Zongyi Guo^3^,** **Linyuan Shen^1,2,*^,** **Li Zhu^1,2,*^**

^1^ Key Laboratory of Livestock and Poultry Multi-omics, Ministry of Agriculture and Rural Affairs, College of Animal and Technology, Sichuan Agricultural University, Chengdu 611130, China.

^2^ Farm Animal Genetic Resource Exploration and Innovation Key Laboratory of Sichuan Province, Sichuan Agricultural University, Chengdu 611130, China.

^3^ Chongqing Academy of Animal Science, Chongqing 402460, China.

*** Correspondence:**

Linyuan Shen^1,2,*^

shenlinyuan@sicau.edu.cn

Li Zhu^1,2,*^

zhuli@sicau.edu.cn

**† These authors contributed equally to this work.**

**Supporting information**

**Figures**

**
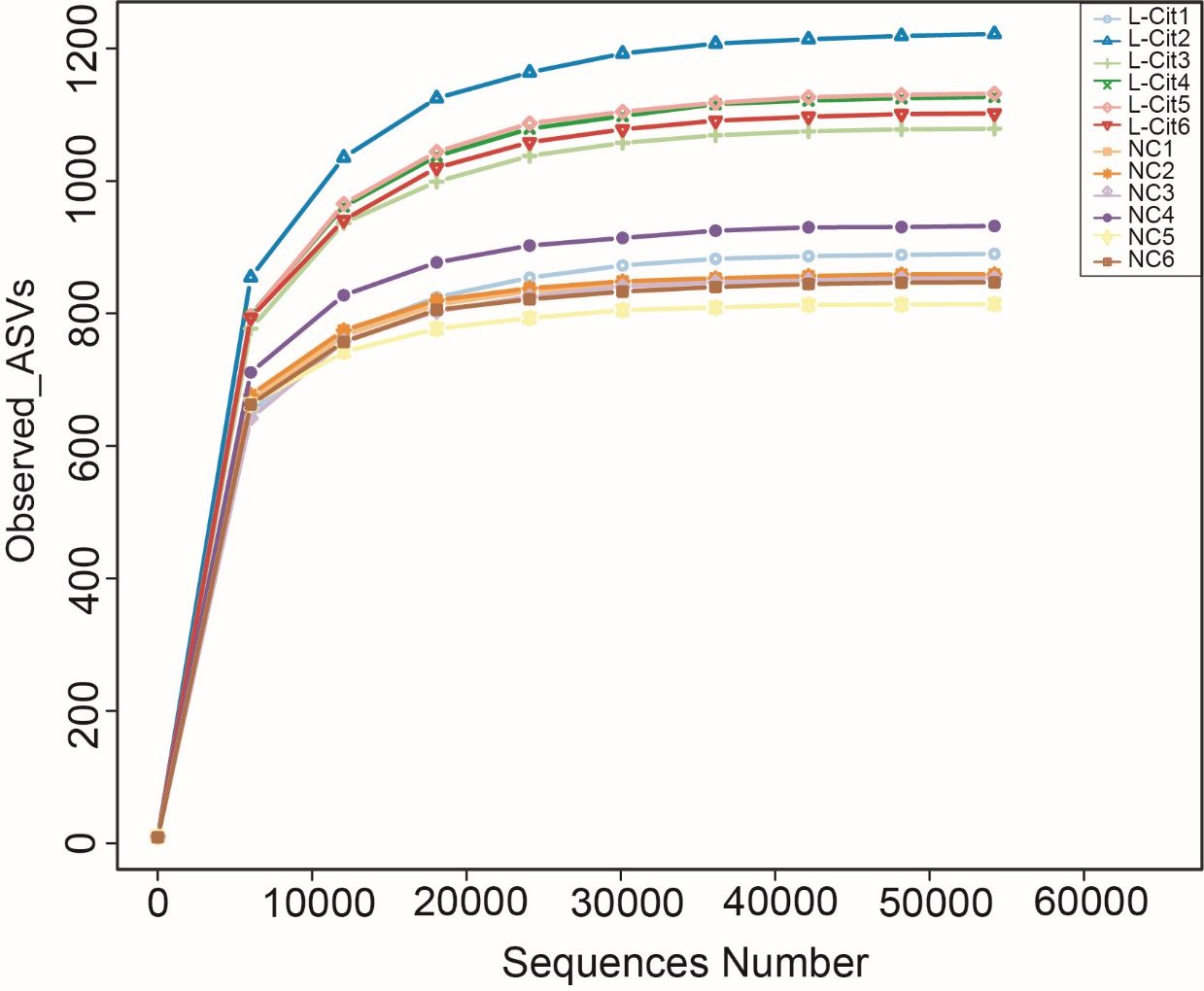
**

**Supplementary Figure 1.** The dilution curve of all samples.

**
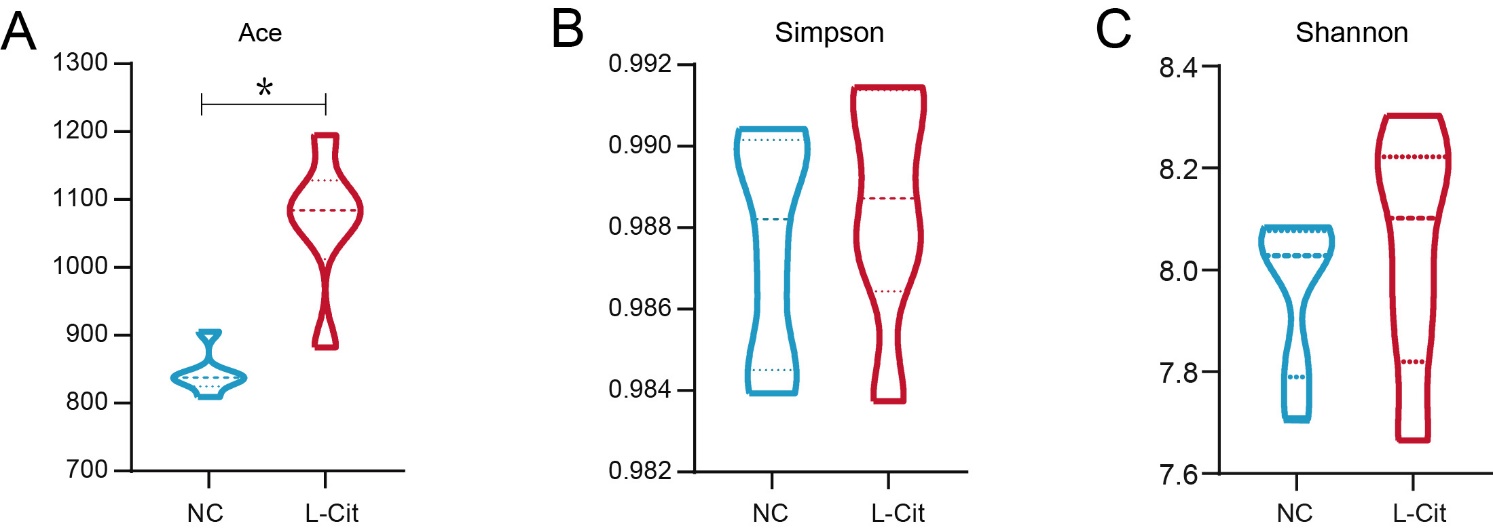
**

**Supplementary Figure 2.** (**A**) Ace index. (**B**) Simpson index. (**C**) Shannon index.


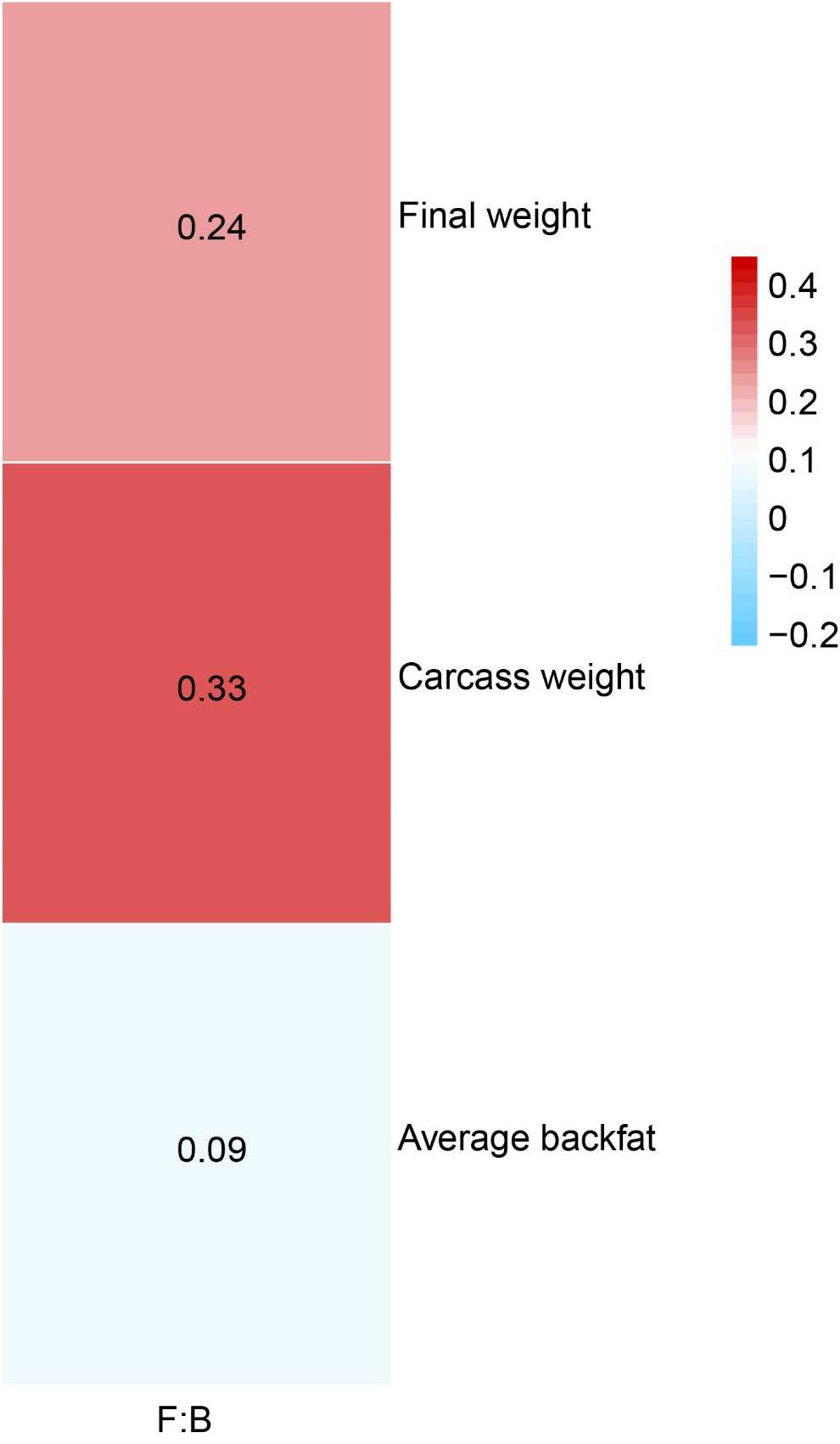


**Supplementary Figure 3.** Correlation analysis of Firmicutes : Bacteroidetes (F:B) ratio and final weight, carcass weight and average backfat.


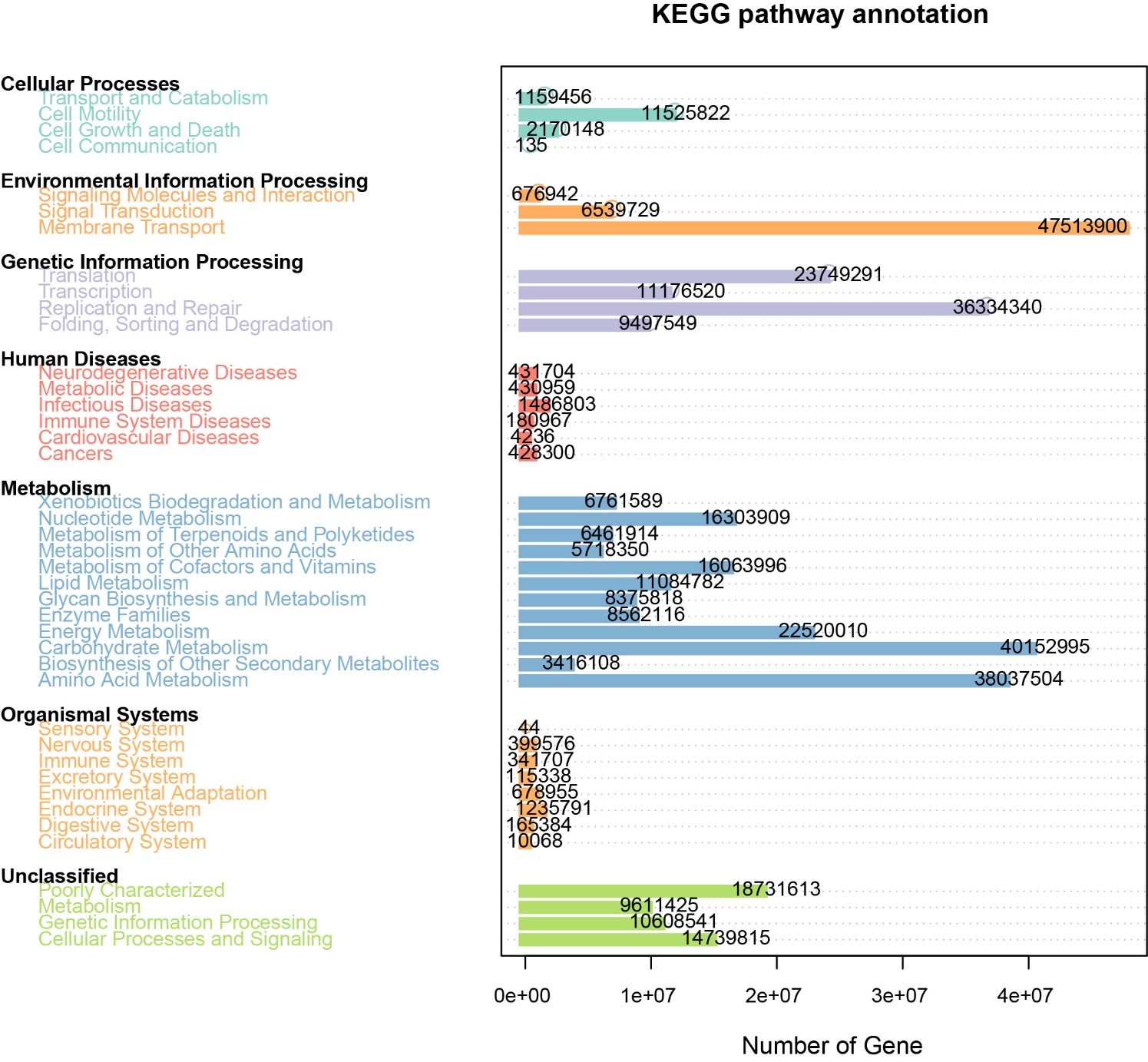


**Supplementary Figure 4.** The number of non-redundant genes assigned to KEGG pathways.
