# Supplementary material for: Effects of dietary L-Citrulline supplementation on growth performance, meat quality, and fecal microbial composition in finishing pigs
Source: Front Microbiol. 2023 Aug 3;14:1209389. doi: 10.3389/fmicb.2023.1209389 (PMC10442155; doi:10.3389/fmicb.2023.1209389)
Supplement: Supplementary file 2 [file Supplementary_Table_1.docx]

**Supplementary Table1**

The composition and nutrient levels of the basal diet.

| Feed ingredient | Nutrient levels | Content（%） |
| --- | --- | --- |
| Corn | Crude protein | ≥14.0 |
| Flour | Crude fiber | ≤8.0 |
| Wheat | Crude ash | ≤8.0 |
| Soybean meal | Calcium | 0.40-1.20 |
| Stone powder | Total phosphorus | ≥0.40 |
| Calcium hydrogen phosphate | Sodium chloride | 0.30-0.80 |
| Sodium chloride | Lysine | ≥0.65 |
| Vitamins and vitaminoids | Water | ≤13.0 |
| Mineral elements |  |  |
| L-lysine |  |  |
| Threonine |  |  |
| Mold inhibitor (calcium propionate) |  |  |
